# BIRTH COHORT DIFFERENCES IN BURDEN OF MULTIMORBIDITY OVER TIME AMONG CHINESE OLDEST-OLD ADULTS

**DOI:** 10.1093/geroni/igae098.0821

**Published:** 2024-12-31

**Authors:** Junmin Zhu, Yafei Wu, Siyu Duan, Ya Fang

**Affiliations:** Xiamen University, Xiamen, Fujian, China (People’s Republic); Xiamen University, Xiamen, Fujian, China (People’s Republic); Xiamen University, Xiamen, Fujian, China (People’s Republic); Xiamen University, Xiamen, Fujian, China (People’s Republic)

## Abstract

**Objective:**

Multimorbidity has significantly burdened healthcare systems worldwide. Understanding the longitudinal changes of multimorbidity over time among the oldest-old adults is necessary but limited. This study aimed to investigate the birth cohort effects on multimorbidity burden among Chinese oldest-old adults and compare the differences across two decades of longitudinal surveys.

**Methods:**

Data from the Chinese Longitudinal Healthy Longevity and Happy Family Study were utilized. Due to the high mortality and attrition rates in the oldest-old adults, we included 2603 and 2404 participants aged 80 and older in 1998 and 2008 surveys, respectively, with each survey followed up for a decade of repeated assessment. The burden of multimorbidity, defined as the presence of 12 chronic health conditions, was analyzed. The generalized linear mixed models were employed to evaluate the birth cohort effects. Additionally, we analyzed the impact of demographic, lifestyle, and socioeconomic factors on the burden of multimorbidity.

**Results:**

In the 1998 longitudinal survey, the earlier birth cohorts had a higher multimorbidity burden over time than those born later (β = 1.217-1.335). Conversely, in the 2008 longitudinal survey, the older birth cohorts showed a lower multimorbidity burden over time (β = 0.631-0.777). The burden of multimorbidity was influenced by gender (female), residence (urban), region (eastern of China), and smoking history.

**Conclusion:**

Our findings revealed that the higher burden of multimorbidity among the Chinese oldest-old individuals has shifted from the older birth cohorts to the more recent ones, highlighting the urgent need for comprehensive policies promoting healthy ageing and early intervention measures.

